# The Relationship of Spiritual Health with Quality of Life, Mental Health, and Burnout: The Mediating Role of Emotional Regulation

**Published:** 2018-01

**Authors:** Mehdi Akbari, Sayed Morteza Hossaini

**Affiliations:** 1 Department of Clinical Psychology, Faculty of Psychology and Education, Kharazmi University, Tehran, Iran.; 2 Quran and Hadith Research Center, Baqiyatallah University of Medical Sciences, Tehran, Iran.

**Keywords:** *Burnout*, *Emotional Regulation*, *Mental Health*, *Quality of Life*, *Spiritual Health*

## Abstract

**Objective:** The World Health Organization's definition of health now stands open to severe criticism due to changes in today's world and the accompanying mental void; in addition to physical, psychological, and social aspects, spiritual health and its interaction with the other aspects has been studied in scientific literature and recent research. The present study was conducted to investigate the mediating role of emotional regulation in the relationship between spiritual health with quality of life, psychological health, and burnout.

**Method** **:** In this study, 231 staff from Baqiyatallah University of Medical Sciences completed Spiritual Well-Being Scale (SWBS), Difficulties in Emotion Regulation Scale (DERS), World Health Organization Quality of Life-BREF (WHOQOL-BREF), General Health Questionnaire-28 (GHQ-28), and Maslach Burnout Inventory (MBI). The gathered data were analyzed using Pearson correlation, Hierarchical Regression analysis, and Sobel’s test.

**Results:** All variables were correlated with one another (p<0.001). The hierarchical regression analysis and Sobel’s test indicated that the emotional regulation have a relative mediating role in the relationship between spiritual health and quality of life (ß=0.53, Z=4.05, p<0.001) and a complete mediating role in the relationship between spiritual health with mental health (ß=0.68, Z=5.62, p<0.001) and burnout (ß=0.70, Z=6.12, p<0.001).

**Conclusion: **There is a complex and non-linear relationship between spiritual health and the areas of quality of life, mental health and burnout. This relationship is potentially influenced by emotional regulation.

Following extensive industrial developments and urbanization, communities have witnessed the spread of many psychiatric disorders, which are among the most important determinants of the risk of common non-communicable diseases including cardiovascular disease, cancer, diabetes, and a large number of chronic physical illnesses(1). The World Health Organization's definition of health now stands open to severe criticism due to changes in today's world and the accompanying mental void; in addition to physical, psychological, and social aspects, spiritual health and its interaction with the other aspects has been studied in scientific literature and recent research(2).

Spiritual perspective on beliefs, attitudes, values, and behaviors has effects on the biochemistry and physiology. These effects on mind and body are called spiritual health (3). Although it has been more than 5decades since the term “spiritual health” has been proposed, the aspects of this very important pillar are still unknown and that is the reason why recent researches try to clarify the complex and multi-dimensional concept of spiritual health and to ingrain this concept as the fourth component of the concept of health with scientific support (3).

The review on related research literature revealed that among the variables related to this structure, quality of life, mental health, and job burnout have the greatest association with spiritual health (1-3).

In the 2 last decades, the relationship between quality of life and spiritual health has been emphasized. The result of Allahbakhshian Farsani et al.’s research (4) on multiple sclerosis patients indicates that quality of life in patients who find a meaning for life based on spirituality during illness has improved compared to their quality of life at the time of diagnosis. The existence of a relationship between spiritual health and quality of life has been confirmed in several studies. The researches of Borchardt et al. (5) on evaluation of chronic diseases, Bossing (7) on study of cancer patients, and Fisch et al. (8) on study of different conditions have supported this idea. In study of D’Almeida et al. (9) on African-American female AIDS patients, there was a positive relationship between being healthy and physical and mental aspects of quality of life, and they concluded that spirituality was a very important factor in quality of life of African-American female AIDS patients (9). Basavaraj et al. (10), by reviewing quality of life in AIDS patients, showed psychological well-being, social supporting systems, dealing with different conditions strategies, and spirituality are important predictors of quality of life in these patients (10).

Spirituality in work is an attempt to make sense of transpersonal, interpersonal, and intrapersonal connections in personal career to achieve self-development in humanity and divinity excellence (11, 12). Another study illustrates that spirituality in an organization have positive effects on creativity, job satisfaction, team performance, and organizational commitments (3). Another study on a financial company found that there is a very impressive and positive relationship between spirituality of workers and their job satisfaction (13). Many studies have focused on the effect of spirituality on work environment. In this context, some factors can be mentioned, such ashonesty and trust (14), organizational commitment (15), decreasing the willingness to leave work, increasing job satisfaction (16), and increasing creativity and productivity (17). Kingereskey and Sckripeng (15) have expressed that workers with higher spiritual health are more loyal to the organization and are more commited to their duties, so spiritual health is the best predictor for job performance.

The connection between mental health and spirituality has come to the interest of many psychologists. Many studies (18-21) have indicated that spiritual health has an impressive effect on mental health. Doolittle and Farrell (19) in a study conducted on Christian patients found that rate of depression was lower in patients who have higher spiritual health. Queunig, George, and Peterson (22) studies showed that spiritual health predicts faster depression recovery, particularly in patients who have not improved their physical function (22). In an extensive analysis which reviewed 850 studies on the relationship between religious/spiritual thoughts and different aspects of mental health, many studies showed that if people have spiritual health they will experience better mental health and will adapt to stress more successfully (23). In another analysis of 350 studies, it was found that people with higher spiritual health have healthier bodies; healthier life styles, and need less medical care (24). In another research, Disroziers and Miller (25) indicated that daily spiritual experiences and clemencies resulted in lower depression rate. Bahrami (26) observed the effects of group spiritual training on female students. Aliani and Mahin (27) study among 369 students showed that anxiety level will decrease dramatically with more praying.

The literature suggests that spiritual health has a strong relationship with quality of life, mental health, and job burnout. However, the major question is whether spiritual health is linked to these variables, or are there other variables which play a mediating role in this regard. By reviewing researches, it was found that one of the concepts that have been added in practical and clinical fields is emotion regulation in terms of psychological pathology, psychiatric disorders treatment, job performance, and even general population issues in developmental psychology (28, 29). Grass and Thompson (28) identify emotion regulation as one of the used strategies to decrease, increase, suppress or maintain excitement and believe that emotion regulation is one of the intrinsic characteristics of humans. Role of this variable has been studied in several researches recently as mediating variable in aforementioned fields (29). Several studies illustrated the relationship between emotion regulation and spiritual intelligence. Results of VanLyon and Kelsoler (34) and Alkins and Cavendish (35) researches revealed that spiritual health and emotional intelligence are highly correlated. Their researches have shown that spiritual intelligence helps emotional intelligence to grow and that emotional intelligence help people earn higher emotional intelligence, and thus live a joyful life with physical and mental health, spiritual health and no stress (36). In Iran, Raghibi et al. (37) examined the relationship between emotional and spiritual intelligence in compatible couples before their marriage and concluded that emotional and spiritual intelligence has a better condition in compatible couples (37). In a study on the students of Isfahan University of Medical Sciences, Hajian et al. found a significant relationship between spiritual health and emotional health (38).

Given the importance of spiritual health as a related structure associated with psychological variables and the importance of emotion regulation, this study aimed at investigating the mediating role of emotion regulation in the relationship between spiritual health with quality of life, mental health, and job burnout.

This cross-sectional study was conducted to review the direct and indirect effects (mediatory effect) of set of variables. The study sample consisted of employees of Baqiyatallah University of Medical Sciences. Sampling was done using availability method. Study samples participated in this survey willingly and all of them have been gifted a training package, consisting of a notebook and apencil. The response time for each set of inventories was 25 minutes. Inclusion criteria were as follow: age 18 to 65 years, willingness to participate, no evidence of severe psychiatric disorders, and not experiencing adverse events such as loss of a loved one, physical illness, accident, etc. Considering population size and using Cressi and Morgan’s table (39), with 95% of trust rate and 10% loss rate, 250 inventories were distributed and by eliminating incomplete inventories, 231 inventories (118 females and 113 males) entered the final analysis. The procedure of the study was that all participants were informed of the study’s aims and provided written informed consent before completing the battery of questionnaires, which were administered in rotated order to control order effect. After signing an informed consent, the participants were provided with a package of questionnaires. Furthermore, participants were assured that their demographic information would be kept confidential. Also, they were informed that they could leave the study at any time. The following tools were used to collect the data. The inventories were presented to participants in random order.

## Materials and Methods

*Spiritual Well-Being Scale (SWBS)*

This test was made by Palotzin and Alisson (40) in 1982 and includes 20 questions and 2subscales. Odd questions are related to the religious well-being subscale and examine people’s experience of a satisfying relationship with God, and even questions are related to existence of a well-being subscale, which tests the goal- oriented sense and life satisfaction. Palotzin and Alisson reported the Cronbach's alpha coefficients of religious well-being, existential well-being, and the total scale to be0.91, 0.91, and 0.93, respectively. The psychometric properties of this scale have been studied by Dehshiri et al. (41). Retested reliability coefficients of the total scale, religious well-being, and existential well-being have been reported to be 0.85, 0.78, and 0.80 respectively. Cronbach's alpha coefficient for the total scale for spiritual well-being is reported to be0.90 and for religious and existential well-being subscales it was 0.82 and 0.87, respectively. Validity of the scale has been calculated thorough factor analysis and correlation with scores of happiness, religiosity, and psychological disorders and has been reported at an acceptable level (41).

*Difficulties in Emotion Regulation Scale (DERS)*

This scale has been designed by Gratz and Roemer in 2004 to measure emotional disorder and emotional self-regulation strategies; it has 36 items scored on a 5- degree Likert scale. Cronbach’s alpha coefficient is reported 0.93 and biweekly retested reliability coefficient is 0.85 (42). The reliability of the Persian version by Asgari et al. through internal consistency is reported to be 0.86 and concurrent validity of the inventory was confirmed by Beck depression scale and Multidimensional Pain Inventory (MPI) (43).

*General Health Questionnaire-28 (GHQ-28)*

This questionnaire was drafted first by Goldberg; it has 60 articles and its short 28- question form was used in this survey. The short form of this questionnaire was made from its long version by Goldberg and Hiller (44). This questionnaire is one of the psychiatric screening tools for the general population and it is used widely to find non-psychotic mental disorders in different conditions (44). This questionnaire has 4subscales to examine somatic symptoms, anxiety and insomnia, social dysfunction, and depression. Validity of this questionnaire by mentioned scales is, respectively, 85%, 74%, and 84%; and it is 92% for the total questionnaire. Reliability coefficients are calculated to be 70%, 93%, and 90%; and 91% for the totalreliability.In addition, the cut-off point of 7 has been considered for diagnosis in each subscale (45). Many researches have been done on the quality of psychometric GHQ-28 in Iran (45-46), and this questionnaire is one of the most reliable means of determining mental health in general population (47).

*Maslach Burnout Inventory (MBI)*

Maslach Burnout Inventory was designed by Maslach and Jackson in 1993 to measure burnout (48). This inventory consists of 22 separate statements measuring3 aspects: (1) sentimental or emotional exhaustion, (2) depersonalization, and (3) personal sufficiency sense. This inventory is based on a 7- degree Likert scale, ranging from 0 to 6. Maslach and Jackson have calculated internal reliability for each subscale; the internal reliability of the inventory by Cronbach’s alpha coefficient and retested coefficient are reported to be 71% to 90%and 60% to 80%, respectively. Hosseiniani and Noferesti (49) have reported the internal reliability Cronbach’s alpha coefficient of 0.84 to 0.93 and retested coefficient of 60% to 80%. Validity of this inventory has beencalculated usingconvergent validity method by a correlation between scores of this inventory and those of Galdard burnout inventory that has been resulted in the correlation coefficient between these 2inventories, which is 0.59. Ramezaninejad et al. (50) have calculated the validity coefficient of this inventory to be 0.78 in their studies using 20 and 22 questions formats of Maslach burnout inventory.

*World Health Organization Quality of Life-BREF (WHOQOL-BREF)*

Quality of life inventory was designed by World Health Organization in 2004 to evaluate quality of life. (51). The short version of this inventory contains 26 questions and evaluates4domains of physical health, mental health, social relationships, and environmental health with 24 questions, the remaining 2 questions evaluate the general domain of quality of life. After calculations, scores from 4 to 20 are achieved for each domain. Nejat et al. (52) have validated this scale and reported the following results: Cronbach’s alpha coefficient for a healthy population in domains of physical health (0.70), mental health (0.73), social relationships (0.55), environmental health (0.84), and biweekly retested reliability coefficient for the total tool (0.70).

Data were analyzed using Statistical Package for the Social Science Version16 (SPSS-16) using Pierson correlation method to study the relationship between the variables of the research. Hierarchical regression analysis and Sobel’s test were used to find the mediating role of emotion regulation in the relationship among spiritual health, mental health, burnout, and quality of life based on the results proposed by Baron and Kenny (53) in the study of mediating variables in psychology. Based on Baron and Kinney’s model (53), we can claim that a variable plays a mediating role in 2 independent and dependent variables when the 4 following conditions are met: (1)standardized regression coefficient beta (ß) between independent variable and the dependent variable is significant; (2) standardized regression coefficient beta (ß) between the independent variable and the mediator variable is significant; (3) dependent variable has significant effect on the mediating variable after controlling the effect of independent variable on dependent variable; and (4) in the third regression equation, standard regression coefficient beta (ß) related to the connection between independent and the dependent variables should completely lose its significance (full mediating role), or if maintaining significance, the amount should decrease dramatically compared to this scale in the first equation (partial mediating role). Sobel’s test should be taken to measure the significance of the mediating role, which is also called indirect effect. This test determines the significance of the mediating role based on zero hypothesis, which assumes that indirect effect coefficient is zero (53).

## Results

In this study, 21.5% of the participants were younger than 30 years, 39.7% between 30 and 40, and 23% were between40 to 50 and about 16% were over 50 years. There were 118 female and 113 male participants. Of the participants, 15.4%were single and 84.6% were married. Of the respondents, 5.7% had high school diploma, 4% were colleague graduates, 46.3% had a bachelor degree, 31.6% amaster degree, and 12.4%adoctorate degree. Of all inventories, 7.6% were incomplete and were put aside list wise. Assumption of normality of every 4scales was studied and confirmed using Kolmogorov-Smirnov test. The Means and standard deviations of self-report measures of the participants are as follow: spiritual health (M= 94.06, SD= 8.98), emotional dysregulation (M= 84.65, SD= 7.76), mental health (M= 28.44, SD= 4.18), quality of life (M= 93.23, SD= 9.09), and burnout (M= 47.56, SD= 6.33).Pierson correlation coefficient results revealed a significant relationship between all variables of the research (Table 1). No multicollinearity (correlation more than 0.75, 49) was observed, so none of the variables was excluded from the final analysis. 

*A. The Relationship between Spiritual Health and Quality of Life: The Mediating Effect of Emotion Regulation*

**Figure 1:**
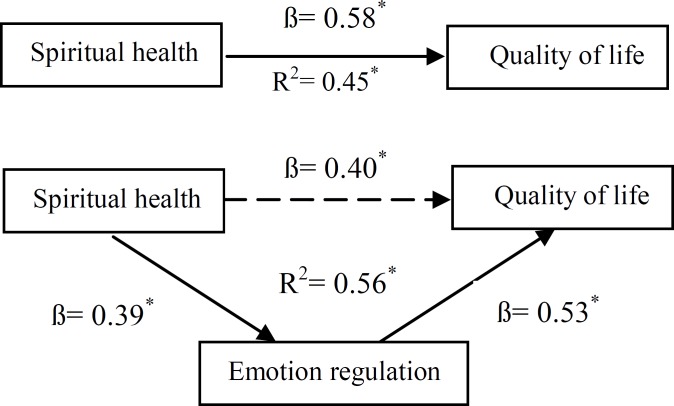
TheMediating role of Emotional Regulation in Relation with Spiritual Health and Quality of Life (Sobel’s test: Z=4.05, p< 0.001)

The first hypothesis of this study implied that emotion regulation plays a mediating role between spiritual health and quality of life using hierarchical regression analysis (Table2). In fact, considering research results, it can be stated that emotion regulation plays all 4conditions of Baron and Kinney’s model to relative intermediation role: (1)standardized regression coefficient beta (ß) of spiritual health and quality of life is significant (p<0.001, ß=0.58); (2)standardized regression coefficient beta (ß) of spiritual health and emotion regulation is significant (p<0.001, ß=0.39); (3)standardized regression coefficient beta (ß) of emotion regulation and quality of life is significant (p<0.001, ß=0.53) after the effect of spiritual health control; and (4)after emotion regulation control, standardized regression coefficient beta (ß) of spiritual health and quality of life maintaines its significance and its amount decreases from 0.58 to 0.42. Sobel’s test results (Z=4.05, p<0.001) supports the finding that emotion regulation has a partial mediating role in the relationship between spiritual health and quality of life (Figure 1).

*B. The Relationship between Spiritual Health and Mental Health: The Mediating Effect of Emotion Regulation*

**Figure 2:**
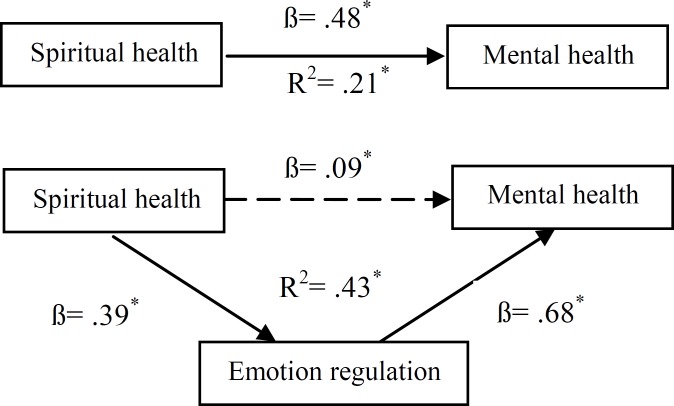
TheMediating Role of Emotional Regulation in Relation with Spiritual Health and Mental Health (Sobel’s test: Z=5.62, p< 0.001)

The second hypothesis of this study implied that emotion regulation has played a mediating role in the relationship between spiritual health and mental health using hierarchical regression analysis (Table 3). The results indicated a full mediating role in the relationship between spiritual health and psychological health: (1)standardized regression coefficient beta (ß) of spiritual health and mental health is significant (ß=0.48, p<0.001); (2) standardized regression coefficient beta (ß) of spiritual health and emotion regulation is significant (ß=0.39, p<0.001);(3)after controlling the role of spiritual health, standardized regression coefficient beta (ß) of mental health and emotion regulation is significant (ß=0.68, p<0.001); and (4)after controlling the role of emotion regulation, standardized regression coefficient beta (ß) of spiritual health and psychological health is not significant and its amount decreases from 0.48 to 0.09 (ß=0.09, p>0.001). Sobel’s test results (Z=5.62, p<0.001) supports the finding that emotion regulation has a full mediating role in relation between spiritual health and mental health (Figure 2).

*C. The Relationship between Spiritual Health and Burnout: The Mediating Effect of Emotion Regulation*

**Figure 3:**
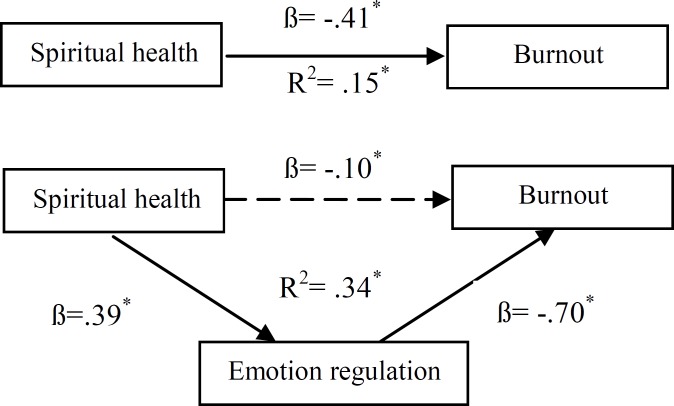
TheMediating Role of Emotional Regulation in Relation with Spiritual Health and Burnout (Sobel’s test: Z=6.12, p< 0.001)

The first hypothesis of this study suggested that emotion regulation plays a mediating role between spiritual health and burnout using hierarchical regression analysis (Table 4). In fact, according to the results, it can be stated that emotion regulation plays a full intermediating role between spiritual health and burnout: (1) standardized regression coefficient beta (ß) of spiritual health and burnout is significant (ß=-0.41, p<0.001); (2)standardized regression coefficient beta (ß) of spiritual health and emotion regulation is significant (ß=0.39, p<0.001); (3)after spiritual health control, standardized regression coefficient beta (ß) of emotion regulation and burnout is significant (ß=-0.70, p<0.001); and (4)after emotion regulation control, standardized regression coefficient beta (ß) of spiritual health and burnout is not significant (ß= -0.10, p>0.001). Sobel’s test results (Z=6.12, p<0.001) supports the finding that emotion regulation has a full mediating role in relation between spiritual health and burnout (Figure 3).

**Table 1:** Correlation Matrix of the Research Variables

**Variables**	**1**	**2**	**3**	**4**
Spiritual health	1			
Emotional dysregulation	- 0.52*	1		
Mental health	0.46*	- 0.57*	1	
Quality of life	0.67*	- 0.71*	0.57*	1
Burnout	- 0.39	0.52*	- 0.27	- 0.40

* A significant correlation at the 0.001 level.

**Table 2:** The Results of Hierarchical Regression and Sobel’s Test for the Mediating Role of Emotional Regulationin Relation with Spiritual Health and Quality of Life

**Dependent V.**	**Independent V.**	**Total R** ^2^	**F**	**B**	**SE**	**Beta**	**Sobel’s test (z)**
Quality of life	Spiritual health	0.45	16.45	2.01	0.21	0.58*	4.05^*^
Emotion regulation	Spiritual health	0.27	11.23	2.95	0.71	0.39*	
Quality of life	Spiritual health	0.56	22.12	1.58	0.18	0.42*	
	Emotion regulation			0.86	0.09	0.53*	

* A significant at the 0.001 level.

**Table 3:** The Results of Hierarchical Regression and Sobel’s Test for the Mediating Role of Emotional Regulation in Relation to Spiritual Health and Mental Health

**Dependent V.**	**Independent V.**	**Total R** ^2^	**F**	**B**	**SE**	**Beta**	**Sobel’s test (z)**
Mental health	Spiritual health	0.21	9.51	1.94	0.43	0.48*	5.62*
Emotional regulation	Spiritual health	0.27	11.32	2.95	0.71	0.39*	
Mental health	Spiritual health Emotional regulation	0.43	18.31	0.32	0.29	0.09*	
				1.17	0.02	0.68*	

* A significant at the 0.001 level.

**Table 4:** The Results of Hierarchical Regression and Sobel’s Test for the Mediating Role of Emotional Regulation in Relation to Spiritual Health and Burnout

**Dependent V.**	**Independent V.**	**Total R** ^2^	**F**	**B**	**SE**	**Beta**	**Sobel’s test (z)**
Burnout	Spiritual health	0.15	8.56	1.05	0.32	-0.41*	6.12*
Emotional regulation	Spiritual health	0.27	11.32	2.95	0.71	0.39*	
Burnout	Spiritual health Emotional regulation	0.34	14.46	0.61	0.31	-0.10*	
				1.45	0.05	-0.70*	

* A significant at the 0.001 level

## Discussion

The main purpose of this research was to study the mediating role of emotion regulation in the relationship between spiritual health and quality of life. Analysis of the research data revealed that based on Baron and Kinney’s model, emotion regulation has a partial mediating role in the relationship between spiritual health and quality of life. In addition, by controlling the emotion regulation, the standardized regression coefficient beta (ß), which is related to spiritual health and quality of life, was weakened significantly. Thus, its quantity decreased from 0.58 to 0.42, indicating a relative mediating role between spiritual health and quality of life. This finding shows that the direct effect of spiritual health on quality of life is more impressive than the indirect effect of this system by emotion regulation. This finding indicated a strong connection between spiritual health and quality of life (4-8).Results of mentioned researches have clarified that religious and spiritual beliefs and activities as supporting or mediating agents can reduce life stress and promote quality of life by giving hope and make life meaningful. They can also affect every aspects of people’s life physically, psychologically, and socially. Considering the positive connection between spirituality and quality of life and the proper role of spirituality in quality of life prediction, strengthening spirituality, religious aspects, and encouraging people to do the related rituals, we can promote quality of life. The research results also illustrated that spiritual health has an indirect effect on quality of life through emotion regulation. Results of the former studies indicate that emotion regulation has an important role in quality of life. Emotion and excitement have a key role in different aspects of life like adapting to life changes and stressful events, so emotion regulation is a basic principle in initiation, evaluation, and organization of adaptive behavior and also prevention of negative excitements and maladaptive behaviors, leading to have better quality of life. Hence, spiritual heath with effects on emotion regulation and using adaptive emotion regulation strategies probably leads to promotion of quality of life for everyone (54-57).

The second objective of the research was to study the mediating role of emotion regulation between spiritual health and mental health. Results of the current study support the full mediating role of emotion regulation between spiritual health and mental health. In other words, by controlling the role of this variable, standardized regression coefficient (ß) associated with the relationship between spiritual health and mental health, lost its significance and its value dropped from 0.48 to 0.09. Results of meta-analysis studies (61) indicate that spiritual health is more effective on mental health than physical health. Psychological, social and behavioral fields have been influenced by spirituality. Well-being, hope, optimistic view, being goal- oriented, strong will, feeling in control, self-control, and adaptation with different stressors have reported to be higher in people with spiritual beliefs than other people (61).Many years ago, it was known that feeling guilty would cause psychological disorders, especially depression, in spiritual people. However, reviewing the results of many studies disproves this idea (19). The results of another research showed that emotion regulation strategies can play a mediating role in pregnant women's mental health, such that adopting certain negative strategies for emotional regulation, such as rumination and catastrophic thinking, was associated with incidence of emotional problems and psychological disorders and decline in sleep quality in pregnant women (61).Also, the interesting result was the impressive and strong role of emotion regulation variable between spiritual health and mental health. In other words, the greatest impact of spiritual health on mental health is by emotion regulation. The Fifth Edition of Diagnostic and Statistical Manual of mental disorders (DSM-5) (62), shows that more than 50% of axis 1 clinical disorders, more than 90% of axis 2 personality disorders, and in sum more than 75% of psychological disorders are related to emotion regulation (63). In cases such as mood and anxiety disorders, the problems are so extensive that all the disorders are defined based on dysregulated emotions. The influence of problem with emotion regulation is obvious in other cases, such as borderline personality disorder, post-traumatic stress disorder, alcohol and drug abuse, and some other disorders. It indicates that emotion regulation problems are main reasons for an abnormal psychology and that emotion regulation is a key to treatment (64). People with psychiatric disorders experience many difficulties related to emotions such as lack of emotions understanding, more negative reactions to emotions, and inability to favorably change the negative emotion (65). In fact, several years ago, theorists like Teasdale (66) have suggested that prone and non-prone people to depression are different not by their primitive response to a negative event, but by their ability to correct (improve) a negative feeling caused by that event. Thus, based on this study and previous researches, it can be concluded that spiritual health can improve mental health by its effects on emotion regulation.

The third purpose of this study was to investigate the mediating role of emotion regulation in the relationship between spiritual health and burnout. The results clarified that emotion regulation in the relation between spiritual health and burnout has played a full mediating role. Standardized regression coefficient (ß) associated with the relationship between spiritual health and burnout lost its significance, and its value declined from 0.41 to 0.10 by controlling the role of this variable. This result is consistent with findings of various studies (14-17) about correlation between spiritual health and burnout. Despite the strong correlation between these 2variables (spiritual health and burnout), results of this study support the indirect effect of spiritual health on burnout and the full mediating role of emotion regulation. This finding also confirms former researches (67-69) about the strong relationship between emotion and burnout. Emotion in organizations and organizational psychology had been neglected because everyone taught that organizations are rational-logical matters in general. However, the efforts of psychologists, who realize the importance of excitement and emotion in behavior and attitudes in organizations, put this matter in serious challenges and questions (67). The review of the issue was mainly about job satisfaction and motivation and did not include obvious emotions. Later researchers gave more legitimacy to this issue by analyzing the reason of importance of emotion and excitement. Meanwhile, addressing this issue was associated with fluctuations. One of the most recent advancements in the field of emotions in organizations is affective events theory (AET), proposed by Weiss and Cropanzano in 1996 (67). Main concepts of this theory are as follow: (1)work behavior of an employee is mainly determined by he/she feels at the time; (2)work environment is one of affective events sources, which generates these feelings; and (3)emotional responses of the employees determine their attitude and later reactions. In AET, behaviors can be emotion-driven, whether negative behaviors like anger and violence, or positive behaviors like self-driven help or expressing job satisfaction. The important behavior determined by attitudes which indicates more mature mood. This behavior could lead to more effective work or antisocial or charitable activities (68-70).The former researches confirmed the role of spiritual health on decreasing job burnout by creating hope, honesty, and trust, promotion of organizational commitment, reducing the desire to leave job, and increasing creativity and productivity. However, recent findings found that the strong relationship between spiritual health and burnout was highly influenced by emotion regulation strategies, and this new finding can open a new horizon to improve the efficiency of organizations’ employees. Several emotional coping strategies had either a significantly helpful or harmful impact on emotional exhaustion and depersonalization as indices of burnout. Acceptance and active coping were strongly associated with lower emotional exhaustion and depersonalization. Conversely, self-blame, denial, disengagement, and humor were associated with higher emotional exhaustion and depersonalization (71).Gallo de Moraes et al. (72) investigated the impact of emotional intelligence on burnout syndrome, professional satisfaction, compassion, and communication skills of trainees in the field of intensive care unit. In their research, 26 trainees participated, with an average emotional intelligence of 158, while 23 suffered from burnout syndrome. The results revealed that high emotional Intelligence correlated with low rates of emotional exhaustion.

## Limitation

The present study had several limitations that should be considered in interpreting the results. One of the limitations of this study was to perform this research on limited and identified population (university staff) and generalizing the results to general population based on these results should be done with caution. Another limitation was that the subscales of process and outcome variables were not studied due to the amount of sample. In addition, another limitation of the present study was that the sample of this study had more religious characteristics, which reduced the generalizability of the results. Finally, the findings were obtained in a descriptive-correlation design and causal deduction was not possible. Thus, considering these limitations, it is suggested that similar studies be performed with a larger sample size and that subscales of the process and outcome variables be studied. It is also suggested that this research be repeated in the form of a causal design.

## Conclusion

overall, based on the results, it can be concluded that although spiritual health is one of the most important predictors of quality of life, mental health, and burnout, the mediating role of emotion regulation in this relationship signified the importance of this new and practical concept. Replication of this study with larger sample size and on the general population will help generalizing data. Reviewing these subscales will deepen our knowledge about the causalrelationships between these variables. Review and evaluation of the research tools’subscales may lead to more clear and more accurate information about mediating role of the used variables. Moreover, it is suggested that futurestudiesbe performed in the form of a design of structural equation model and causaldeductionto achieve a theoretical model.
